# On a New Compound Gas, Resulting from Animal Decomposition Taking Place in the Living Body: With Some General Remarks on Tympanitis. Being the Substance of a Memoir Read before the Royal Society

**Published:** 1822-03

**Authors:** 


### ANIMAL CHEMISTRY.

Art. V.—*On a new Compound Gas, resulting from Animal Decomposition taking place in the living Body: with some general Remarks on Tympanitis. Being the Substance of a Memoir read before the Royal Society.* By the Editor.

The subject, to which I take the liberty of calling the attention of the Society, appears to me, from various reasons, to possess no inconsiderable share of interest.

The existence of a combination of sulphur and azote, forming a peculiar aëriform fluid, has been maintained, by several chemists, such as FOURCROY, GIMBERNAT, SCHAUB, MANHEIM, REAUMONT, and, recently, by LAUSBERG. Fourcroy imagined that, by burning sulphur in azote, he had succeeded in obtaining the gas in question: other chemists thought they had detected it in mineral waters. Its presence, however, in the animal economy has never been mentioned, as far as I know, by any preceding writer. In announcing the existence of this gaseous compound in the living body, as the result of animal decomposition, I shall chiefly confine myself to a statement of facts, and to the enumeration of those inferences to which the facts themselves have necessarily led me.

A girl, aged thirteen, was admitted, in June 1817, under the following circumstances, into the Hôpital des Enfans at Paris; where, through the kind friendship of Mr. JADELOT, the principal physician, and the liberality which distinguishes the public arrangement of the hospitals of that metropolis, I had the opportunity of visiting, daily, upwards of five hundred cases of infantile diseases, for which beds are provided in that excellent establishment.—Mademoiselle Dubancour's person was developed with somewhat more than usual precocity for her age. Originally of a sanguineous and melancholic temperament, she had, from the moment which marked her years of puberty, suffered either from irregular menstruation or a lowness of spirits. Her eyes were deeply sunk within the orbits; the head ached incessantly and violently. She felt frequent nausea; became subject to periodical vomiting; and an insidious fever, with its night vigils and chilling perspirations, preyed on her enfeebled constitution. There was a short hectic cough, which, with some other circumstances, would have led a physician to consider her case as one of pulmonary consumption; but the appearance of other symptoms sufficiently indicated that she was affected by a very different complaint. The menstrual discharge had long since ceased to appear; excruciating pains, and a general lassitude, followed; until, unable any longer to support the weight of her difficulties, the poor patient applied for relief at the above-mentioned hospital.

A few days after her admission, it was discovered that the abdomen had gradually swelled beyond its natural size. The skin was tense, hot, and painful. The tumor was uneven in its surface. On applying the hands to it, fluctuation could distinctly be felt. Some attributed the tumefaction to air; others, to a collection of both air and fluids: the event proved that the latter conjecture was correct.

It is needless to relate to what treatment the patient was subjected, and to detail the progress of the complaint, during the short period of her existence. Mlle. Dubancour sunk gradually, and died a month after her admission into the hospital. The body, according to the practice in the French hospitals, on most occasions, was carefully examined. I was charged with the operation; and, as there was no doubt in my mind but that, besides a liquid of some kind, we should find an aëriform fluid in the abdominal cavity, great attention was paid to the mode in which both were collected, so as to exclude the admixture of any extraneous substance.

Mr. Berenger, one of the house-pupils *(élève interne)* of the hospital, who had kindly assisted me in the examination, delivered to me the gas and fluid in the same vessel in which they had been received, for the purpose of their being analyzed. I likewise procured a sufficient quantity of the matter, which the patient had frequently rejected from her stomach, during the last days of her illness. On these three fluid substances, the following experiments, some days afterwards, were instituted.

1. *Preliminary Operation.*

My first object was to separate the gas from the fluid, and to free the former from a considerable portion of the green matter which it held in suspension, and which gave, also, a predominant character to the fluid in question. The operation, necessary to effect this, was performed without any sensible loss, or diminution of volume, in the gas. The whole quantity, thus procured, measured ninety-six cubic inches; that of the fluid amounted to about four pints.

2. *Examination of the Gas.*

A. One hundred parts = 1 cubic inch, were repeatedly washed in lime-water; when an absorption was observed to take place = 15, and a precipitate to follow, which dissolved in acetic acid with effervescence, indicating a quantity of carbonic acid present in the whole of the gas $= \frac{96 \times 15}{100} = 14.40$ cubic inches. A lighted taper being now introduced, it was instantly extinguished; the flame being, at the same time, *blown out* of the tube. The same result had attended a similar experiment, made before the washing of the gas in lime-water.

B. One hundred parts of the gas, freed from all the carbonic gas by experiment A, were exposed to the action of caustic potash for several hours. No absorption took place: a fact which excludes the idea of the gas being sulphuretted hydrogen, as the peculiar nature of the smell would have induced us to suspect.

C. To the gas, B, one hundred parts of oxygen were added; and the mixture was exposed to a succession of sparks from an electric machine. Neither inflammation nor detonation took place: but an absorption was observed = 10, corresponding to a quantity of sulphurous acid, which was found on admitting water into the eudiometer.

D. By the successive introduction of small portions of hydrogen into the gazeous mixture, C, until inflammation was no longer visible, the remaining quantity of the oxygen, employed in experiment C, was got rid of. The volume of hydrogen consumed in this operation, and measured each time it was introduced, was found to be = 180; giving, for the quantity of oxygen with which it had been made to combine, a number = 90, corresponding with the quantity that had remained after the absorption noticed in experiment C.

E. The remaining gas, having been subjected to the usual experiments, was ascertained to be pure azote.

F. To prevent all doubts with regard to the cause of the absorption observed in experiment C, which might, else, have been ascribed to the presence of some very minute portion of hydrogen, I added a known proportion of that gas to 100 parts of the mixture under examination, and proceeded to burn it, by means of oxygen and electricity. The result was a condensation equal to the quantities of the two gases employed, and no more. It is needless to observe, that far different would the case have been, had any hydrogen been present besides that employed in the experiment.

From the above experiments, we may conclude that the gazeous mixture contained in the cavity of the abdomen, consisted of

| | | |
|---|---|---|
| Sulphuretted azote . . . . | 85 | = 100 |
| Carbonic acid gas . . . . . | 15 | |

### 3. *Chemical Analysis of Sulphuretted Azote.*

It has been seen that, when one hundred measures of oxygen were employed to analyze the gazeous mixture, (experiment C,) an absorption = 10 had taken place, corresponding to a quantity of sulphurous acid formed during the operation. Now, as 100 cubic inches of oxygen will burn 33.75 grains of sulphur to form the acid in question, the absorption, which occurred in experiment C, will necessarily indicate the presence of 2.52 of sulphur for every 100 cubic inches of azote; and, as 100 cubic inches of azote are said to weigh 29.25 grains, it follows that the weight of sulphuretted azote in our case will be = 31.77, giving these proportions :

| | | |
|---|---|---|
| Azote . . . . | 92.03 | = 100. |
| Sulphur . . | 7.97 | |

But one hundred measures of azote appear, in the present instance, to unite to sulphur, and to form the gas in question, without undergoing any change of volume: hence, sulphuretted azote must be composed of 1 proportion of sulphur,
8 proportions of azote.

4. *Physical and Chemical Characters of Sulphuretted Azote.*

A. It is colourless, permanently elastic, transparent.

B. Perfectly insoluble in water, and not condensible by cold.

C. Not a supporter of combustion.

D. Has a peculiar smell, approaching to that of sulphuretted hydrogene, yet perfectly distinct.

E. It does not suffer any alteration or decomposition by electricity.

F. It does not possess any marked alkaline or acid properties.

G. Tastes like animal matter in an incipient state of putrefaction.

Having ascertained the curious fact of such a gas existing, under very peculiar circumstances, in the living animal system, it was natural for me to feel desirous of knowing from what source that gas took its origin. In order to attain, more satisfactorily, my object, I next proceeded to the examination of that portion of the serous fluid contained in the abdomen, which had been separated from the gas; and I think I am enabled to draw, from the results of my inquiries, a series of inferences, which, together with an account of the analysis of the fluid in question, I beg leave to communicate to the Society.

Among the maladies that afflict mankind, none is more frequent than inflammation. Every part of the human economy, with scarcely any exception, is susceptible of being inflamed; particularly in the female economy, which, from constitutional disposition, seems still more obnoxious to this peculiar morbid alteration.

In almost all cases of inflammation, there is an effusion of one or more of the constituent principles of the blood, either in some of the cavities, or in the cellular texture of the inflamed part. This fact, well known to physiologists, I have myself had occasion to ascertain in upwards of fifty cases, within the last four years; and it is according to the particular principle which has been separated from the blood, under such circumstances, that inflammation terminates either in simple effusion or in suppuration. Why one of the constituent principles of the blood should be separated from that fluid, during inflammation, rather than another, so as to lead to a different termination of the disease, according to the principle so secreted, is a question which I am not prepared to discuss, much less to answer. My business is to offer facts, rather than to hazard theories. Whenever effusion follows inflammation, water and albumine have been the only principles found to have been separated during that morbid process. When, on the contrary, the fibrine of the blood has, also, been detected in the effused serum, suppuration has constantly followed. Gangrene obtains when the secreted fluid undergoes the process of putrefaction.

It is not one of the purports of this paper to say any thing of the two latter modes of termination, in cases of inflammation. I wish to confine myself solely to the first, in as far, only, as it may bear on the main object of my present inquiry.

When, through any morbid cause, inflammation takes place in some part, or parts, of the animal system, especially in or near one of its cavities, if the effused fluid, which so frequently accompanies it, consists only of a portion of the water and albumine of the blood, we may hope to obtain, by an appropriate treatment, either its absorption or its evacuation. If neither of these terminations be quickly brought about, the quantity of the effused fluid (whether its relative quantity remains stationary, or continues to increase,) will destroy, more or less rapidly, first the functions, next the vitality, of the parts affected. This is what we witness, in cases of inflammation of the lungs, or of the pleura, or of the pericardium; and, likewise, in inflammation of the serous membrane lining the cavity of the abdomen, particularly in females. In the two first species of inflammation, when absorption (or resolution, which is the same thing,) fails, suffocation, and consequently death, may ensue, owing to the quantity of the effused fluid present in the cavity of the thorax. In the third species, the motions of the heart are gradually impeded, from the same cause; which likewise vitiates, or altogether suspends, the functions of the abdominal viscera, when the peritoneal covering is inflamed.

In about twenty cases of inflammation of the latter kind, which proved fatal, and which came within my knowledge during the last three years, I have had an opportunity of analyzing the effused fluid; and I never could discover any other constituent principle of the blood in it than water and albumine, with a small proportion of common salt. Wurtzer, Vanmons, Fourcroy, Vauquelin, and Dr. Bostock, I believe, obtained similar results. But it is not, perhaps, equally known that, when the fluid here alluded to, separated from the blood during the inflammatory action, has, in consequence of defective absorption, sojourned any length of time in one of the cavities above mentioned, or in any part of the cellular tissue, without destroying the life of the individual, a decomposition of one or both of its constituent principles (namely, water or albumine,) not unfrequently takes place, through some peculiar process of nature, directed, no doubt, to the laudable end of removing the disease; but giving rise, at the same time, to new productions and combinations, and constituting tympanitis and emphysema.* This I believe to be the case in most abdominal dropsies of long duration; the decomposition taking place sooner or later, according to circumstances. In support of this opinion, I confidently refer to the case I have related in the present memoir, in which it is shown, in a very manifest manner, that the albumine of the serum effused during the inflammatory disease by which the patient had been affected during life, was gradually decomposed; and that, from such a decomposition, arose the gas, which it has been the more prominent object of this paper to describe. The proofs of this assertion will be found in the following:

5. *Chemical Examination of the Fluid contained in the Cavity of the Abdomen, and secreted during high Peritoneal Inflammation.*

A. The fluid was of a muddy greenish-yellow colour, somewhat resembling whey, with an abundant sediment of a whitish, tenacious, and fatty substance. It was slightly viscid, and became but indistinctly frothy after long agitation.

B. It had a most infectious and strongly ammoniacal smell.

C. It reddened litmus paper and the syrup of violets.

D. Heated in a platina crucible, it gave out a considerable quantity of carbonic acid gas, with effervescence.

E. After a quarter-of-an-hour's exposure to heat, a thin coagulum was formed, which, when cut through, exhibited numerous small cavities. The borders of the capsule became greasy.

F. The coagulum showed evident marks of being alkaline.

G. By incineration, subcarbonate and muriate of soda, with some phosphate of lime, were found both in the coagulum and sediment.

H. Submitted to a calculated analysis, the fluid seemed to consist of

| | | |
|---|---|---|
| Water . . . . . . . . | 90 | = 100 |
| Albumine . . . . . . | 03.5 | |
| Mucus . . . . . . . . | 02.5 | |
| Salts (including loss) . . | 04 | |

These experiments tend to prove, that the fluid in question contained but a very small portion of albumine; a circumstance which must not be lost sight of, as tending materially to confirm

* What chemistry has, in the present instance, detected and explained, ancient writers in medicine, who have also been accurate observers, have ventured to suppose or to conjecture, without the assistance of any chemical knowledge. Thus, SENNERTUS, (in his lib. iii. part iv. sect. ii. cap. 4, *De Tympanite,*) discussing the probable mode in which the accumulation of gas takes place in the cavity of the abdomen, observes, "Nos statuimus aliquam humoris serosi partem, a calore viscerum circumjacentium in vapore resolvi, qui cum exitum non inveniant, in abdomine cumulantur, idque distendunt."

the explanation I have offered of the formation of the gas under consideration.

According to BERZELIUS, the albumine of serum is, to that of the water contained in it, as 8 : 90; and the many analyses of fluids effused during inflammation, recorded in different books, seem to warrant the adoption of these proportions. In our case, therefore, there is a quantity of albumine = 4.5 to be accounted for; and to say that this quantity was decomposed, and produced sulphuretted azote and carbonic acid, (or, in other words, the gaseous mixture examined in this paper,) cannot, I apprehend, be received as a preposterous proposition. Indeed, it will be found that, in all the analyses of animal fluids, secreted during inflammatory action, which have been published, circumstances have, invariably, been mentioned, which indicated that a partial decomposition of the albumine had taken place, as has been the case in the instance I have recorded. In twenty such analyses by Fourcroy, the consistency, colour, &c. of the secreted fluids he examined, varied constantly, according to the length of time during which those fluids had sojourned in the cavities of the body. The proportion of the constituent principles of the effused fluid was seldom the same; the albumine, *especially*, never having been found in the same proportion. The sulphur, too, which has been detected by the several chemists who have analyzed the morbid fluids in question, demonstrate still further the correctness of my supposition. But its combination with azote escaped their notice, because, I presume, having once, by the usual tests, ascertained the presence of sulphur, its more common combination with hydrogene was immediately taken for granted; and the existence of sulphuretted hydrogene gas, in the cavities of the human body, tacitly admitted.

In pursuing further my inquiry, it is curious to see how the elements of albumine may arrange themselves, during its decomposition, so as to give rise to precisely the new gaseous production which I have detected in the case under consideration. For, the quantity of hydrogene present in albumine being, according to GAY-LUSSAC and THENARD = 7.5, we may suppose it to have combined with 2.70 of the azote, to form the ammonia found in the liquid removed from the cavity of the abdomen. The remaining quantity of azote, = 13, will then be equal to the proportion of that principle which was found in the new gas, combined with the whole quantity of sulphur known to exist in albumine, on the authority of the same chemists.

Not so readily, however, can we reconcile the proportions of carbone and oxygene contained in albumine, (according to Gay-Lussac and Thenard,) with the quantity of carbonic acid found in the gaseous mixture, as well as in the liquid which I examined: for, if we suppose all the oxygene of albumine (23.87,) to have combined with 12.20 of the carbon, to form carbonic acid, there will still be an excess of carbon, (40.6,) the absence of which cannot be accounted for: unless, indeed, we suppose it to have combined with other animal matter; a conjecture, by the bye, which I am disposed to entertain, when I reflect on the peculiar morbid appearances which the neighbouring parts presented at the time, and which they are known to exhibit after inflammation.

It will be adding to the authenticity of all the preceding facts, to state that Mr. Barruel, operative chemist to the Faculty of Paris, a gentleman whose skill and accuracy in analytical chemistry is well known, as well as his experience in inquiries of this nature, which he has pursued for upwards of thirty years without interruption, had the goodness to repeat, at my request, the experiments detailed in this paper; and that he obtained similar results, as I find by looking over his notes, which he has been kind enough to commit to my charge.

I have since made albumine the subject of comparative experiments, placing it under various circumstances, and favouring its decomposition by various means and contrivances, in hopes of deriving some knowledge with regard to the formation of the new gas. But, as I have nothing very decisive on the subject, to which I can call the attention of the Society, I shall not, of course, waste their time by the enumeration of all those attempts, which, unfortunately, my present occupations preclude me from prosecuting further.

From the facts and observations brought forward in this paper, I think I am warranted in concluding—

1st. That there exists a combination of sulphur and azote, forming a peculiar compound gas, which may be called *sulphuretted azote.*

2dly. That *sulphuretted azote* may be the result of animal decomposition taking place in the living body.

3dly. That the albumine of serous fluids, secreted during inflammatory action, is often decomposed, and gives rise to the formation of ammonia, carbonic acid, and *sulphuretted azote.*

Lastly. That, by a due consideration of the above circumstances, there is every reason to suppose that means might be devised, calculated to obviate many of the fatal effects arising from the decomposition of certain fluids in the animal economy during disease.

*Saville-row; February* 1822.

